# Community Structure and Survival of Tertiary Relict *Thuja sutchuenensis* (Cupressaceae) in the Subtropical Daba Mountains, Southwestern China

**DOI:** 10.1371/journal.pone.0125307

**Published:** 2015-04-30

**Authors:** Cindy Q. Tang, Yongchuan Yang, Masahiko Ohsawa, Arata Momohara, Si-Rong Yi, Kevin Robertson, Kun Song, Shi-Qiang Zhang, Long-Yuan He

**Affiliations:** 1 Institute of Ecology and Geobotany, Yunnan University, Kunming 650091, China; 2 Key Laboratory of Three Gorges Reservoir Region’s Eco-Environment, Ministry of Education, Chongqing University, Chongqing 400045, China; 3 National Centre for International Research of Low-carbon and Green Buildings, Chongqing University, Chongqing 400045, China; 4 Graduate School of Horticulture, Chiba University, 648 Matsudo, Chiba 271–8510, Japan; 5 Institute of Medicinal Plant Cultivation of Chongqing, Nanchuan 408435, China; 6 Tall Timbers Research Station and Land Conservancy, 13093 Henry Beadel Drive, Tallahassee, Florida 32312 United States of America; 7 Department of Environmental Science, East China Normal University, No. 3663 North Zhongshan Road, Shanghai 200062, China; 8 Dabashan National Nature Reserve, Chengkou 405900, China; Wuhan Botanical Garden, Chinese Academy of Sciences, Wuhan, China, CHINA

## Abstract

A rare coniferous Tertiary relict tree species, *Thuja sutchuenensis* Franch, has survived in the Daba Mountains of southwestern China. It was almost eliminated by logging during the past century. We measured size and age structures and interpreted regeneration dynamics of stands of the species in a variety of topographic contexts and community associations. Forest communities containing *T*. *sutchuenensis* were of three types: (1) the *Thuja* community dominated by *T*. *sutchuenensis*, growing on cliffs; (2) the *Thuja-Quercus-Cyclobalanopsis* community dominated by *T*. *sutchuenensis*, *Quercus engleriana* and *Cyclobalanopsis oxyodon*, along with *Fagus engleriana* and *Carpinus fargesiana*, on steep slopes; (3) the *Thuja-Tsuga-Quercus* community dominated by *T*. *sutchuenensis*, *Tsuga chinensis*, and *Quercus spinosa*, on crest ridges. The established seedlings/saplings were found in limestone crevices, on scarred cliff-faces, cliff-edges, fallen logs, canopy gaps and forest margins. The radial growth rate was 0.5-1.1 mm per year. Its growth forms were distorted. It had strong sprouting ability after disturbances. The *T*. *sutchuenensis* population thrives on cliffs where there is little competition from other species because of harsh conditions and rockslide disturbances. It is shade-intolerant but stress-tolerant. Its regeneration has depended on natural disturbances.

## Introduction

Tertiary relict flora represents some of the rarest and most threatened plant species in the world. Understanding the population structure, demographics, and ecological niches of such species is essential both for their modern-day conservation and to discover the mechanisms that have influenced their biogeography and allowed them to persist for millennia.

Refugia of Tertiary relict flora exist in eastern Asia (China, Japan and Korea), southwestern Eurasia, southeastern and western North America [1, 2, 3, 4]. These regions possess a great number of genera in common, while particular species are usually endemic to only one of the four areas [5, 3, 6, 4]. Species richness of Tertiary relict flora differs greatly among refugia, with the greatest diversity occurring in East Asia and the least in southwestern Eurasia [2, 3, 4].

Since southern China was never covered by extensive and unified ice-sheets, owing in part to its mountainous topography [7, 8], the mountains in the region have had a relatively stable long-term environment, now referred to as Pleistocene glacial refugia, harboring many Tertiary relict plants [9, 10, 11]. Today paleoendemic and monotypic taxa thrive in the Yangtze River valley, and survive in highly fragmented areas with small population sizes in specific habitats [12, 13, 14, 15].

The genus *Thuja* (Cupressaceae) is represented in the fossil record dating back to the late Cretaceous. Fossil records reveal a wide distribution in the Northern Hemisphere over geological time, as in the late Cretaceous in Alaska [16], in the Paleocene in Greenland [17] and Ellesmere Island, Canada [18], in the Miocene of the Sikhote Alin Mountains of Russia [19] and from the late Miocene and the Plio-Pleistocene in Japan [20, 21], and in the Plio-Pleistocene of Peary Land, North Greenland [22]. *Thuja* currently comprises five extant species found disjunctly in East Asia and in North America. *T*. *sutchuenensis* Franch. (Yabai or Sichuan *Thuja*) is endemic to the Daba Mountains at the northern fringe of the Sichuan Basin in the Yangtze River valley of China. *T*. *koraiensis* Nakai (Korean *Thuja*) is found in Korea and on the boundary between Korea and northeastern China (Changbaishsan). *T*. *standishii* (Gordon) Carrière (Japanese *Thuja*) is native to Japan. *T*. *occidentalis* L. (northern whitecedar) and *T*. *plicata* Donn *ex* D. Don (western redcedar) grow in eastern and western North America, respectively [23]. *Thuja* disappeared from Europe and high latitudes during the ice ages of the Pleistocene [24]. The ancestral area of *Thuja* had been presumed to be eastern Asia [25]. Both the North Atlantic land bridge and the Bering land bridge have been considered as possible routes for the migration of ancestral populations to North America [25]. A reverse process has been proposed by Peng and Wang [26], who used multiple gene sequences to infer that *Thuja* could have originated in the high-latitude areas of North America in the Paleocene or earlier, with subsequent expansion into eastern Asia over the Bering land bridge. In that case, the two eastern Asia species *T*. *standishii* and *T*. *sutchuenensis* would have a sister relationship, and could have differentiated in the Oligocene or early Miocene.

The first specimens of *T*. *sutchuenensis* were collected from a single location at 1400 m asl in Chengkou County (formerly Sichuan Province, now part of Chongqing Municipality) by the French missionary P. G. Farges from 1892 to 1900 [27] and was named *T*. *sutchuenensis* Franch. in 1899 [28]. Botanical collecting trips to Chengkou by Chinese botanists later in the twentieth century failed to find T. sutchuenensis, *thus it was considered extinct in the wild* [29] and known only by Farges' specimens in various European herbaria [27]. It was the only conifer listed as extinct in the wild on the global red list of conifers [30]. In October 1999 it was rediscovered in Chengkou by a regional team of Chinese botanists near the location where P. G. Farges made his last collections almost 100 years earlier [31, 27]. It had been subject to extremely intensive logging, as local people used the soft, easily-worked wood for home construction, shingles, and other purposes requiring resistance to rot. Remaining specimens are limited to those left in inaccessible limestone formations, mostly on crest ridges or steep slopes at 800–2100 m elevation, and their seedlings are scarce [31, 32, 27]. It is now ranked as an endangered species on the IUCN red list [33]. However, no ecological research has been conducted on the species in its various topographic surroundings and community associations and its mechanisms of persistence have not been identified.

The objective of this paper is to present field data to answer fundamental questions about the species' ecology: (1) What are the spatial distribution patterns and population structure of *T*. *sutchuenensis*? (2) How is its population maintained within the forest community? (3) How do its regeneration dynamics compare with other species of *Thuja* communities? Answers to these questions should contribute to a better understanding of the current status of this Tertiary relict plant and provide insight into its demographic mechanisms and prospects for survival.

## Materials and Methods

### Study area

**Fig 1:**
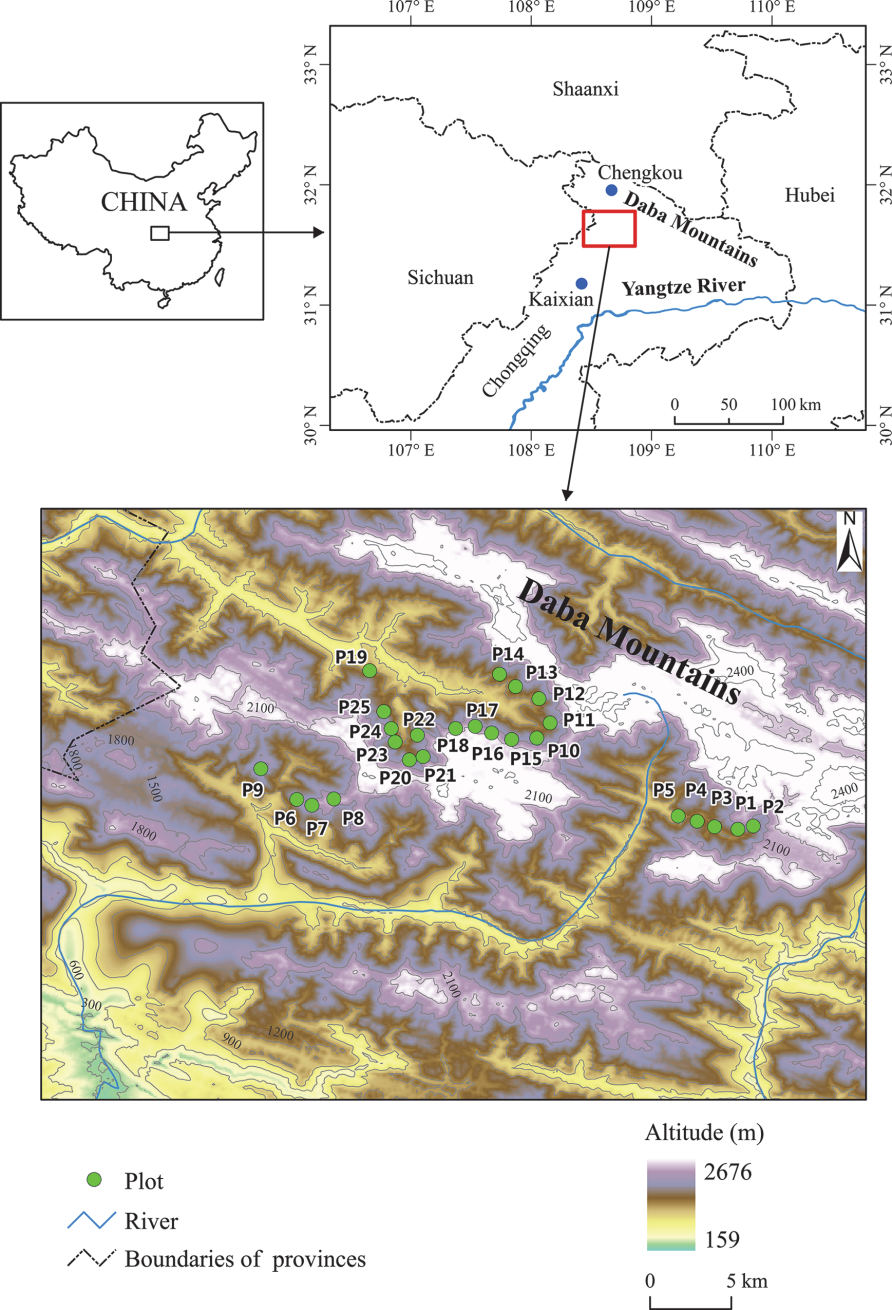
Study sites on the Daba Mountains, southwestern China. The study plots represent the natural distribution range of *T*. *sutchuenensis*.

The study was conducted in the Dabashan Nature Reserve of Chengkou County and the Xuebaoshan Nature Reserve of Kaixian County in the Daba Mountains on the borders of Chongqing Municipality and Sichuan, Hubei and Shaanxi provinces (31°35′52′′–31°41′50′′N, 108° 34′52′′–′ 8°50′34′′E) (Fig 1). During the past century, logging and agricultural development greatly altered the composition and structure of the region's vegetation and landscape. A wide variety of forest types once intermingled at varying spatial scales. Today, the most common vegetation land cover types are even-aged plantations (e.g. *Cunninghamia lanceolata*), early successional plant communities (dominated by *Populus adenopoda* or *Betula utilis*), and particularly coppice forests used for firewood and shrublands regenerated after the abandonment of agricultural fields, comprise the most common vegetation. Embedded within these secondary communities are some remnant forests on the limestone cliffs (inclination: 75°‒90°), steep slopes (inclination: 30°‒75°), and crest ridges (rounded tops of cliffs and slopes), some including stands of *T*. *sutchuenensis*. The steep slopes and crest ridges are partially covered by soils composed of moist and fertile mountain yellow-brown earth. The soil layer is relatively thin and rich in organic matter (2.3–3% of total dry weight), with a thick humus layer (c. 20 cm) and pH of 6.2–7 [27]. The limestone landscape with steep precipices and cliffs is one of the most pronounced topographic features of the Sichuan Basin. The cliffs are especially landslide-prone, being composed of limestone, clay shale, and volcanoclastic and siliceous rock. The rock crevices on the cliffs result from tectonic instability and landslide-prone formations of various rock strata. The main triggering factors of landslides are active faults and rainstorms, especially during May to October [34, 35]. On average, in the range of our study sites there are four landslides per year of various intensities, including cliff rockslides and debris flows [34]. Slope hazard (landslide and collapse) are common in the study area. This is one of the most geologically active regions in China. According to our field observations, the intensity of rockslides or landslides is highest on cliffs, medium on steep slopes, and medium-low on crest ridges. A subtropical monsoon (humid and warm) climate prevails, with a mean annual precipitation of 1114 mm, of which 78% occurs in May–October. The mean relative humidity is c. 73% year-round. Mean annual temperature is 14°C, with 2.7°C in January (coldest month), 24.2°C in July (hottest month).

### Data collection and analysis

Given that *T*. *sutchuenensis* is an endangered species, we obtained permission from the Dabashan and Xuebaoshan National Nature Reserves to conduct the botanical and vegetational survey. In August 2014, we sought out all populations of *T*. *sutchuenensis* in areas where they were known by botanists and local people to exist. We interviewed local villagers to obtain the information on their use of this species. Based locations of populations, we established 25 plots (sizes ranging from 10 × 10 m to 10 × 50 m according to size of the community patch containing *Thuja*) representing the natural distribution range of *T*. *sutchuenensis* in Chengkou and Kaixian counties (Fig 1). Plots were designated as occurring on cliffs, steep slopes, or crest ridges, as described above. We identified all the vascular plant species and recorded diameter at breast height (DBH at 1.3 m tall) and height for all woody plants in each plot. For trees in inaccessible locations such as steep cliff faces, DBH and height were estimated. Young individuals of woody plants 15 cm–60 cm high were counted as established seedlings, and those from 60 cm–1.3 m were counted as saplings. Increment core samples of *T*. *sutchuenensis* were taken at DBH from 31 main stems among the 25 plots in order to estimate size-age relationships. Tree ages were determined using the WinDendro tree-ring image analysis software (version 2003a; Regent Instruments, Sainte-Foy, Quebec, Canada). Analysis of correlation between age and DBH was conducted using DeltaGraph Pro 3.5 software (DeltaPoint, Inc., USA). Plant communities were classified using a floristic similarity dendrogram with Euclidean and Ward’s clustering, using the relative basal area of woody species as the input value (PCORD software [36]). Communities were considered distinct at the 50% floristic similarity threshold. Dominance was determined using a dominance analysis according to relative basal area of each species [37]. The communities were named according to the genera of the first three dominant species. We considered a species to be a major one if it was dominant in at least one community. The Gleason richness index (dGI = S/LnA, S: number of species, A: plot area) [38] was employed to compare species richness among various plots. Differences in species richness among different plant communities were tested using the Tukey–Kramer test for unequal sample sizes. Statistical analyses were performed using STATISTICA software package (StatSoft, USA). Size class and age class distributions of *T*. *sutchuenensis* and its associated major species in various topographic habitats were used to interpret regeneration dynamics.

## Results

### Forest community structure

**Fig 2:**
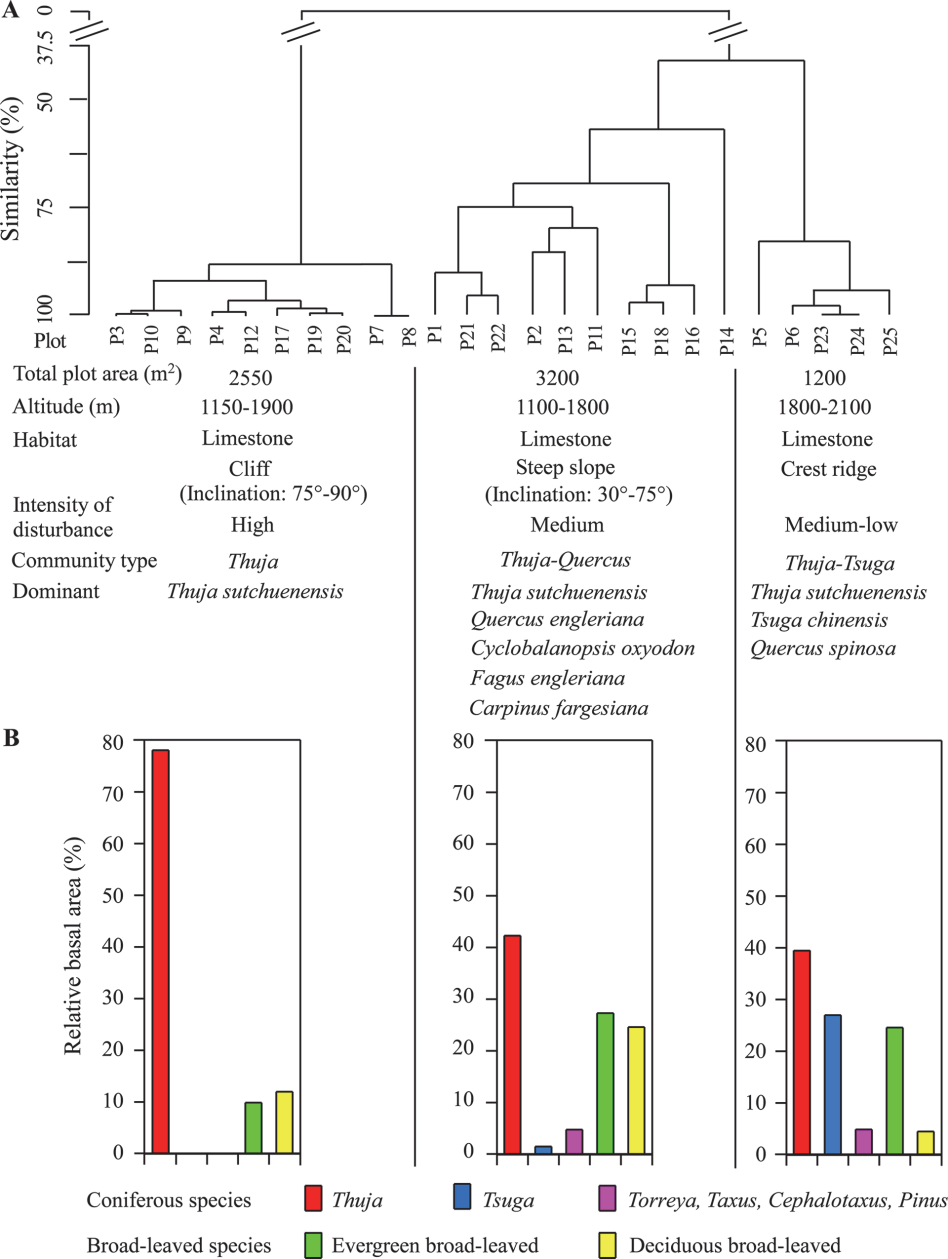
Floristic similarity dendrogram and forest characteristics (A). Relative basal area (%) distribution of species (height ≥ 1.3 m) (B). The evergreen broad-leaved species include the subtropical evergreen broad-leaved species and the evergreen sclerophyllous species of *Quercus*.

**Fig 3:**
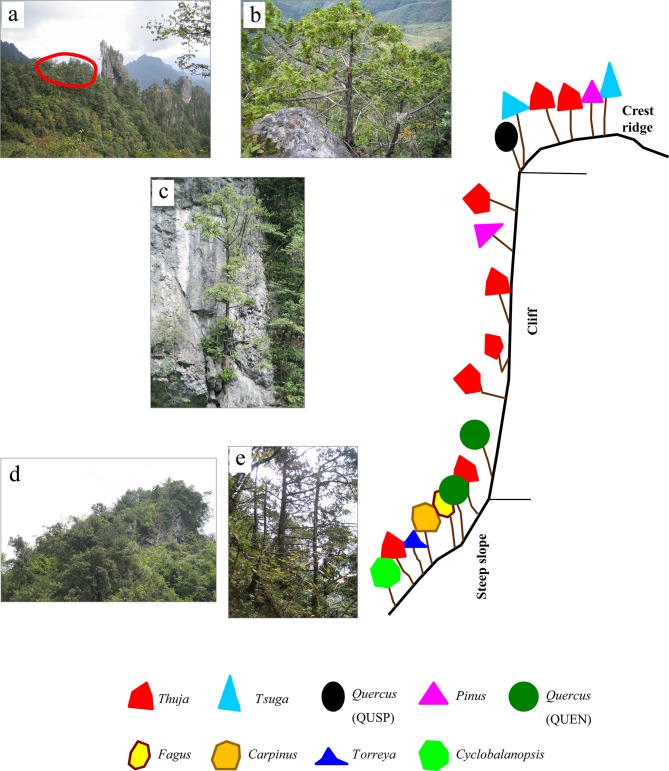
Schematic diagram of the composite representation of distribution patterns of *T*. *sutchuenensis*. The *Thuja-Tsuga-Quercus* community on the crest ridge (a) and (b). The red circled area is shown in (a)-*Thuja sutchuenensis*. A *T*. *sutchuenensis* on the cliff (c). The *Thuja-Quercus-Cyclobalanopsis* community on the steep slope (d) and (e). QUSP: *Quercus spinosa*; QUEN: *Quercus engleriana*.

**Fig 4:**
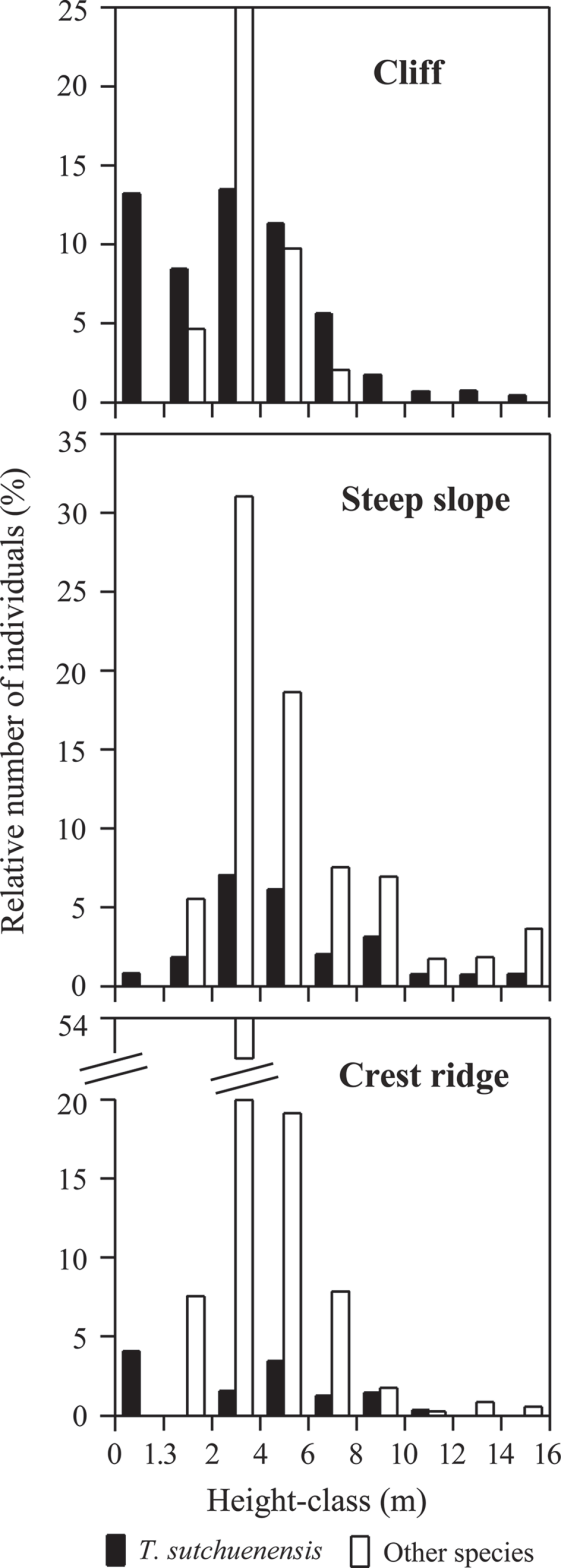
The height-class frequency distribution in different topographic habitats. Bars in the height-class of 0–1.3 m indicate the seedlings/samplings of *T*. *sutchuenensis*.

The dendrogram analysis resulted in classification of the 25 plots into three plant communities corresponding to the three topographic positions (Fig 2A): 1) the *Thuja* community, dominated by coniferous *T*. *sutchuenensis* on cliffs; 2) the *Thuja-Quercus*-*Cyclobalanopsis* community, dominated by *T*. *sutchuenensis*, evergreen sclerophyllous broad-leaved *Quercus engleriana* and subtropical evergreen broad-leaved *Cyclobalanopsis oxyodon* along with some deciduous broad-leaved trees such as *Fagus engleriana* and *Carpinus fargesiana* on steep slopes; 3) the *Thuja*-*Tsuga*-*Quercus* community, dominated by *T*. *sutchuenensis* and another conifer *Tsuga chinensis*, and evergreen sclerophyllous broad-leaved *Quercus spinosa* on the crest ridges. The relative basal area (RBA) of *T*. *sutchuenensis* decreased with decreasing intensity of natural disturbance (rockslide), i.e. from the cliffs to the steep slopes to the crest ridges (Fig 2B). *T*. *sutchuenensis* accounted for 78% of total basal area on the cliffs, 42% on the steep slopes, and 36% on the crest ridges, while *Tsuga* accounted for only 0.1% on the cliffs, 1.4% on the steep slopes, and 31% on the crest ridges. A few other minor conifers, including *Torreya fargesii*, *Taxus wallichiana* var. *chinensis*, *Pinus tabuliformis* var. *henryi*, and *Cephalotaxus fortunei*, grew in all three habitats. Evergreen sclerophyllous broad-leaved *Quercus engleriana* are absent from the crest ridges. Deciduous *Fagus engleriana* existed only on the steep slopes. In total, there were 118 woody species (70 on cliffs, 94 on steep slopes, and 18 on crest ridges) and 37 herbaceous species in the study sites. The major woody species (RBA ≥ 0.5%) composition of each community type is shown in Table 1. The community on the steep slopes had significantly higher Gleason richness (3.73 ± 1.4) than those on the crest ridges (2.27 ± 0.18) and the cliffs (2.78 ± 1.4). A composite representation of the distribution pattern of *T*. *sutchuenensis* and associated major species is shown in a schematic diagram (Fig 3). The height of *T*. *sutchuenensis* reached 16 m, but few exceeded 10 m in all three habitats (Fig 4). The cliffs support the greatest number of *Thuja* seedlings/saplings (15 cm < height < 1.3 m) in the study area (Fig 4).

**Table 1:** Floristic composition for woody species (relative basal area [RBA] ≥ 0.5% in at least one plant community).

Community type		*Thuja*	*Thuja-Quercus-*	*Thuja-Tsuga-*
			*Cyclobalanopsis*	*Quercus*
Topographic habitat		Cliff	Steep slope	Crest ridge
No. of plots		10	10	5
Total plot area (m^2^)		2550	3200	1200
Gleason richness (d_GI_) ± SD		2.78 ± 1.4^a^	3.73 ± 1.4^b^	2.27 ± 0.18^a^
Species	Life form	RBA (%)	RBA (%)	RBA (%)
*Thuja sutchuenensis*	C	**78**	**41.8**	**36**
*Quercus engleriana*	ESBL	3.5	**10.2**	
*Quercus dolicholepis*	ESBL	2.9	0.6	
*Juglans cathayensis*	DBL	2.5		
*Carpinus fargesiana*	DBL	2.3	**3.7**	2.3
*Cyclobalanopsis oxyodon*	EBL	2.3	**4.3**	2.2
*Quercus phillyreoides*	ESBL	1.7	0.7	
*Platycarya strobilacea*	DBL	1.3	3.1	
*Daphniphyllum macropodum*	EBL	1.2	1.7	
*Buxus microphylla* subsp. *sinica*	EBL	0.8		1.4
*Symplocos multipes*	EBL	0.8	0.6	
*Zanthoxylum ovalifolium*	EBL	0.6	0.3	
*Betula austrosinensis*	DBL	0.4		1.1
*Cornus oblonga*	DBL	0.3	3	
*Cyclobalanopsis myrsinaefolia*	EBL	0.2	3.3	
*Emmenopterys henryi*	DBL	0.2	0.5	
*Euptelea pleiosperma*	DBL	0.1	0.5	
*Pinus kwangtungensis*	C	0.2	0.02	0.5
*Viburnum atrocyaneum*	DBL	0.1	0.5	
*Tsuga chinensis*	C	0.08	1.4	**31**
*Pinus tabuliformis* var. *henryi*	C	0.07	0.7	
*Osmanthus armatus*	EBL	0.07	0.2	0.5
*Viburnum betulifolium*	DBL	0.06	0.5	0.07
*Acer davidii*	DBL	0.05	0.5	0.02
*Torreya fargesii*	C	0.03	3.6	4.8
*Taxus wallichiana* var. *chinensis*	C	0.02	1	
*Fagus engleriana*	DBL		**3.7**	
*Quercus spinosa*	ESBL		1.3	**18.1**
*Illicium micranthum*	EBL		0.9	
*Sorbus wilsoniana*	DBL		0.9	
*Ilex* sp.	EBL		0.6	0.009
*Machilus* sp.	EBL		0.5	
*Rhododendron* sp.	EBL		0.2	1.3
*Sorbus glomerulata*	DBL			0.6

Dominant species are indicated by boldface. Significantly different values (*p* < 0.05) of Gleason richness are indicated by different letters by the Tukey–Kramer test. RBA = relative basal area, C = coniferous, ESBL = evergreen sclerophyllous broad-leaved, EBL = evergreen broad-leaved, DBL = deciduous broad-leaved.

Lists of woody species (RBA > 0.01%) occurring in each community type, including their DBH- and height-class frequency distributions, are shown in S1 Table.

### Regeneration dynamics

**Fig 5:**
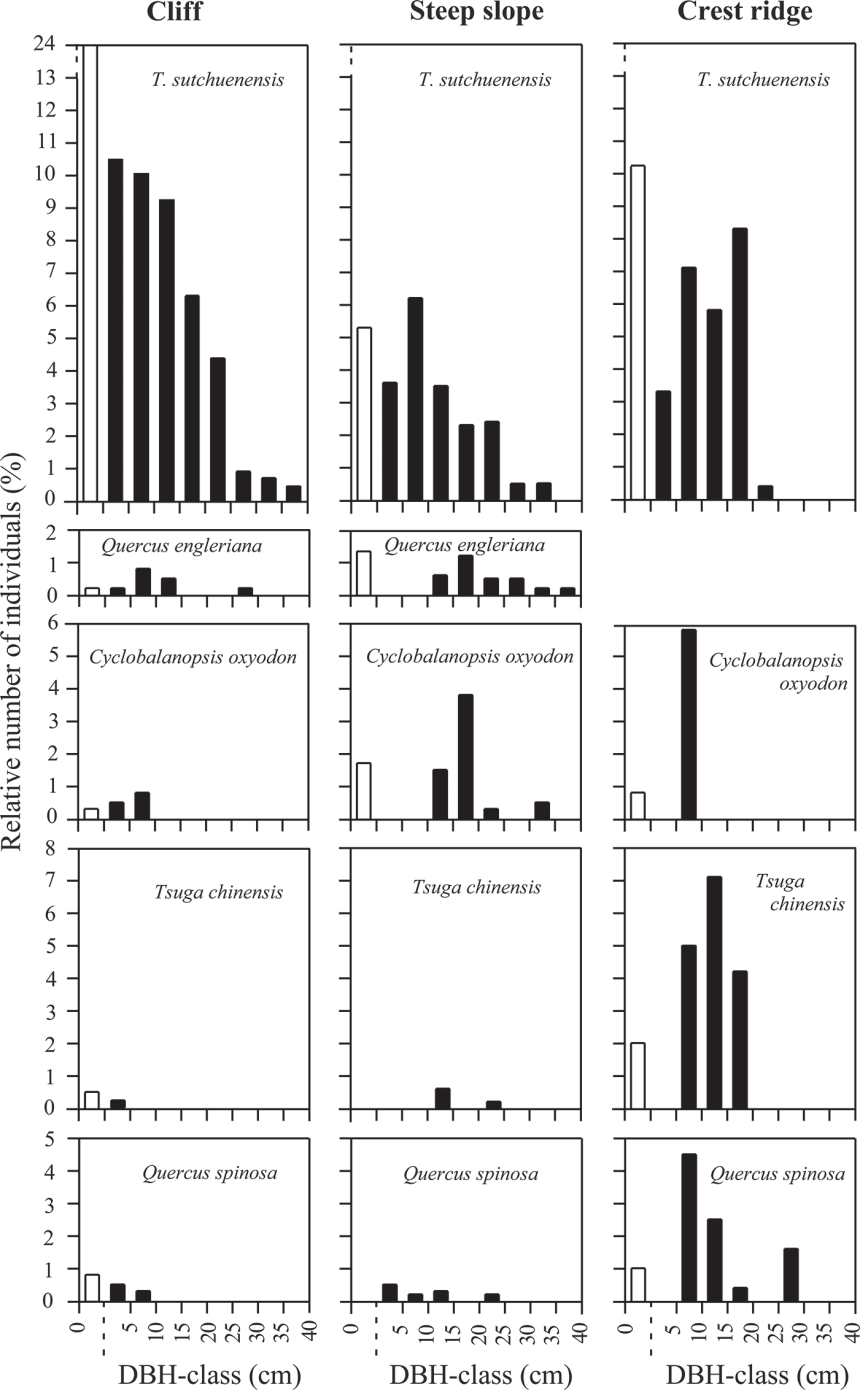
DBH-class frequency distribution for five major species in various topographic habitats. Empty bars indicate the seedlings/saplings.

Size class distributions of the five major species (Fig 5) can be categorized by inverse-J, sporadic, or unimodal types of DBH-size frequency distribution. On the cliffs (*Thuja* community), the DBH of *T*. *sutchuenensis* ranged from 0.5–40 cm and showed the inverse-J type distribution, suggesting that regeneration is active with continual recruitment of seedlings/saplings. Only a few individuals of *Quercus engleriana*, *Cyclobalanopsis oxyodon*, *Quercus spinosa* and *Tsuga chinensis* were present, almost all in small size classes. On the steep slopes (*Thuja-Quercus-Cyclobalanopsis* community), *Thuja* reached 35 cm DBH and showed the unimodal distribution type, suggesting that recent conditions have not been conducive to regeneration. *Quercus engleriana*, *Cyclobalanopsis oxyodon* and *Quercus spinosa* displayed the sporadic distribution type, suggesting periodic regeneration, and *Tsuga* had very few individuals. On the crest ridges (*Thuja-Tsuga-Quercus* community), *Thuja* ranged from 3–25 cm DBH and showed a distribution intermediate between the cliff and slope populations, with *Quercus spinosa* fitting the sporadic type, while *Tsuga* and *Cyclobalanopsis* were of the unimodal type.

### Population age structure

**Fig 6:**
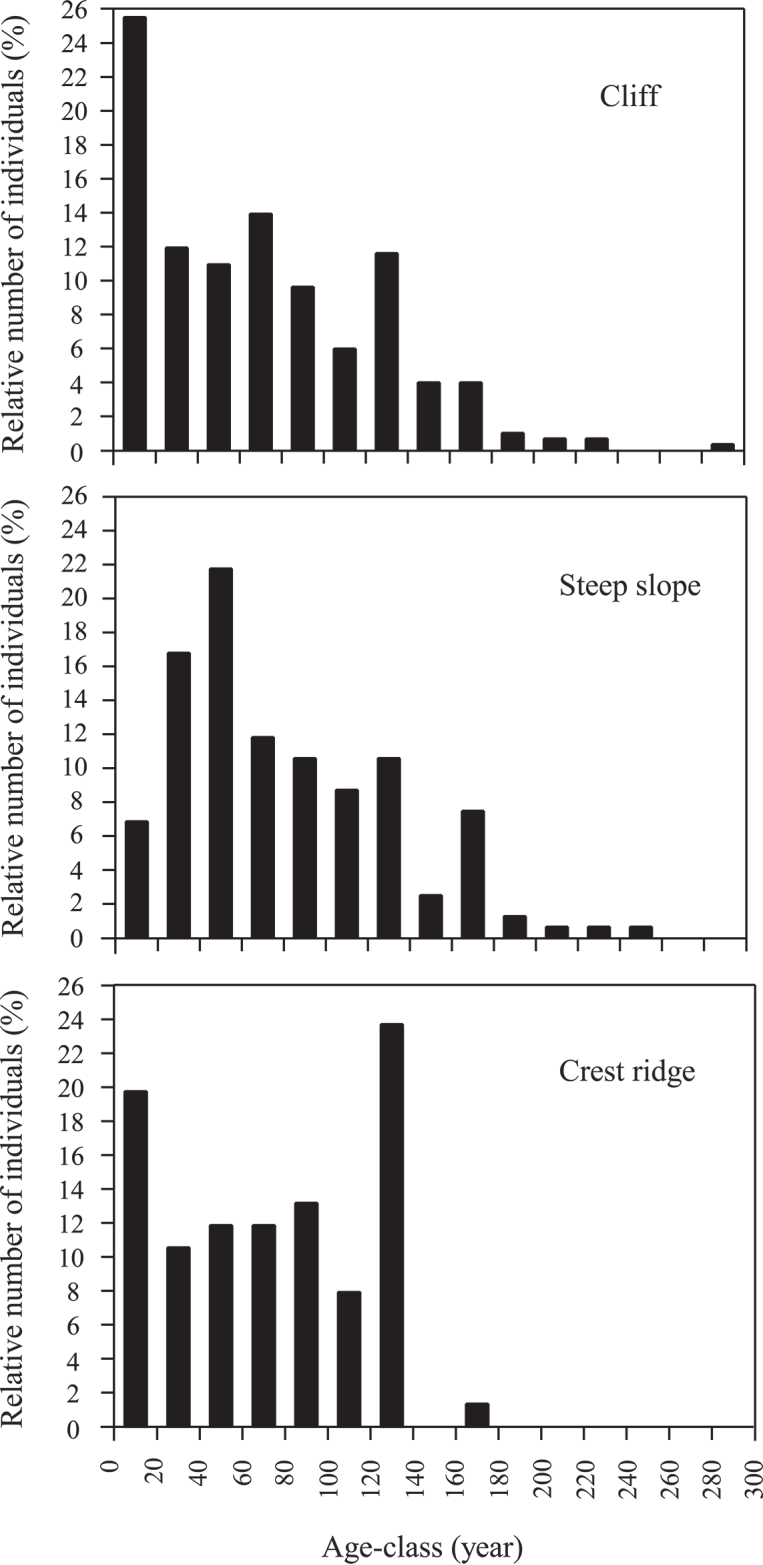
The age structure of the *T*. *sutchuenensis* population in different topographical habitats. Data include individuals ≥ 1.3 m in height and established seedlings/saplings (15 cm< H < 1.3 m).

Age and diameter at breast height at 1.3 m (DBH) for *T*. *sutchuenensis* were fairly strongly correlated (y = 7.1x + 9.03, R^2^ = 0.84, n = 31). The correlation was statistically significant (*p* < 0.001). The growth rate of *T*. *sutchuenensis* was very low. The average radial growth rate per year was 1.1 mm below age 20 years, 0.75 mm from 20–40 years old, 0.5 mm from 40‒90 years old, and it varied between 0.8‒0.5 mm above 90 years. The age structure of *T*. *sutchuenensis* in each topographic habitat is shown in Fig 6. On the cliffs, the age structure suggests continual recruitment with a pattern similar to its DBH-size frequency distribution. On the cliff, nearly 26% of individuals were under 20 years old, indicating better regeneration than in the other two types of topographic habitats, on the steep slopes (7%) or on the crest ridges (19%). The oldest tree (c. 293 years) was located on a cliff. Many older trees with greater DBHs were logged during the past century, based on our observations of logged stumps and our interviews with local villagers.

### Regeneration modes

**Fig 7:**
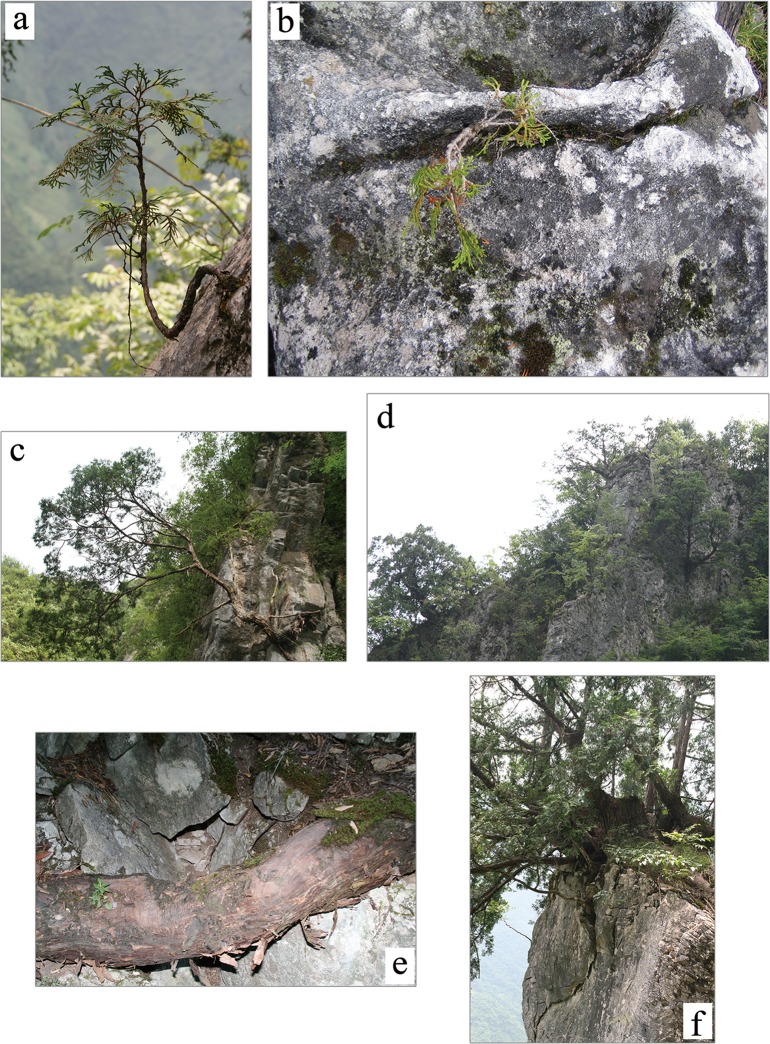
A sapling of *T*. *sutchuenensis* in a crevice on the cliff (a). A seedling of *T*. *sutchuenensis* in a crevice on the cliff (b). The deformed structure of *T*. *sutchuenensis* on the cliffs (c) and (d). A root of *T*. *sutchuenensis* over rocks (e). *T*. *sutchuenensis* resprouting after cutting (f).

Substantial numbers of seedlings/saplings of *T*. *sutchuenensis* were growing on the cliffs (Table 2, see also Figs 4, 5 and 6), but regeneration was generally poor as a whole for all the populations in the three topographic habitats. Most seedlings/saplings were found in rock crevices (Fig 7A and 7B), cliff-faces scarred from rockslides, and cliff-edges where the micro-sites were dry. Some seedlings/saplings were found on fallen logs, canopy gaps, and forest margins by slopes and crest ridges. The stems of *T*. *sutchuenensis* showed evidence of variable disturbances of intensity. Some stems showed human disturbance in the form of cutting, and several adult trees had limbs cut off. However, we saw no evidence of fire disturbance. The growth forms varied; most of the stems were twisted (Fig 7C and 7D). Roots were well developed and widely spread on the outcrops, sometimes reaching over 10 m (Fig 7E). After sustaining damage from natural disturbances (i.e. rockslides or landslides) or cutting, *T*. *sutchuenensis* was able to resprout. The ratio of resprouts to main stems was roughly 15: 1. For example, many individuals on the cliffs had 2–18 resprouts after they were damaged by rockfall, and a main stem of *Thuja* with 140 cm DBH was cut in the 1970s, and now there are 20 resprouts ranging 2–35 cm DBH (Fig 7F).

**Table 2:** Number of seedlings/saplings (15 cm < height < 1.3 m) of *Thuja sutchuenensis* in various micro-sites.

Community type	Topographic	Micro-site	No. of seedlings
	habitat		and saplings
*Thuja*	Cliffs	Rock crevices	42
		Scarred cliff-faces	18
		Cliff-edges	11
*Thuja-Quercus-Cyclobalanopsis*	Steep slopes	Rock crevices	4
		Fallen logs	0
		Canopy gaps	2
		Forest margins	3
*Thuja-Tsuga-Quercus*	Crest ridges	Rock crevices	6
		Fallen logs	2
		Canopy gaps	0
		Forest margins	3

## Discussion

### Growth rates and growth forms

Our results show *T*. *sutchuenensis* to have a slow radial growth rate (0.5–1.1 mm/year) in the topographic context studied, as compared with the other Tertiary relict *Metasequoia glyptostroboides* in its natural habitats in Lichuan, south-central China (0.34–7.81 mm/year, [13]) of Cupressaceae. Individuals of *T*. *sutchuenensis* in existing populations are generally small in stature, but they can grow to be very old. While the oldest tree found (293 years old) was 40 cm DBH, interviews with local villagers and our observations of logged stumps reveal that some stems logged in the 20^th^ century were up to 140 cm DBH, suggesting that they were over 1000 years old. The distinctive traits of stunted and morphologically deformed crowns, slow growth, and exceptional longevity parallel those of *T*. *occidentalis* on exposed cliff edges and faces of the Niagara Escarpment of southern Ontario [39, 40]. On the cliffs of the Niagara Escarpment, the high probability of survival is thought to be linked to slow use of limited resources compensating for slow growth [40]. Cliff-dwelling trees of *T*. *occidentalis* almost without exception have lower growth rates than trees growing in more favorable environments [40]. Biotic interactions on cliffs are low because individuals are widely spaced, competition for light is low or nonexistent, and the root systems of neighboring plants are often completely separated by rock formations [41]. Given the potentially extreme longevity of this species under perennially harsh conditions, it seems that survival is dependent on establishment through the accident of finding safe sites with adequate rooting volume, followed by the lack of any fatal disturbance in that location [42].

### Spatial distribution and regeneration

The altitudinal distribution range of *T*. *sutchuenensis* falls within the subtropical evergreen broad-leaved forest zone and mixed evergreen, deciduous broad-leaved and coniferous forest zone (altitudinal range (800) 1100–2100 m) (Table 1). In contrast, the other four species of *Thuja* grow in the cool temperate and cold temperate forest zones. The distribution, ecological characteristics and major associated species of *T*. *sutchuenensis* as compared with the other four species of *Thuja* are summarized in Table 3. The spatial distribution patterns of *Thuja sutchuenensis* found in this study result from the interactions among natural disturbances, life history traits and stress-tolerance. Marginal habitats formed under conditions of disturbance support distinctive flora and rare species because of differing competitive abilities and tolerance limits among species [57]. The cliffs with high intensity rockslides are solely dominated by *T*. *sutchuenensis*. In contrast, the slopes and crest ridges are dominated by three species capable of forming dense canopies. The size-structures of *T*. *sutchuenensis* and its major associated species, including *Quercus engleriana*, *Cyclobalanopsis oxyodon*, *Tsuga chinensis* and *Quercus spinosa* in the three topographic communities, suggest trade-offs in environmental requirements among those species (Fig 5). Species with narrow regeneration niches often require special sites [58]. Seedlings/saplings of *T*. *sutchuenensis* growing in crevices, on scarred cliff-faces and cliff-edges, on fallen logs, in canopy gaps and forest margins by steep slopes and crest ridges, show that the species' regeneration depends on disturbances which create or maintain these exposed, low-competition conditions (Table 2). Its seeds are minute (1.13g per 1,000 seeds) [59] and can take advantage of wind-dispersal to fall into rock crevices on the cliffs, unlike the big acorns of *Quercus* and *Cyclobalanopsis*. *Tsuga chinensis* is shade-tolerant but susceptible to drought [60], and so unable to become established on the exposed cliffs. Resprouting can also be a strategy in maintaining population stability and continuity, especially in habitats where disturbances that do not kill the whole plant frequently occur. *T*. *sutchuenensis* resprouts vigorously after disturbances that damage stems and branches, suggesting adaptation to such disturbances. *Quercus engleriana*, *Quercus spinosa* and *Cyclobalanopsis oxyodon* also showed resprouting abilities (the mean ratio of resprouts to main stems is 9:1) after disturbances on the cliffs, but we did not find many resprouts for these four species on the slopes and crest ridges. This indicates less frequent natural disturbance on the slopes and crest ridges. *T*. *sutchuenensis* has relatively strong recruitment on the cliffs compared to the slopes and the crest ridges. This also suggests its strong tolerance of xeric sites. The species appears to be the stress-tolerant type as classified by Grime [61].

**Table 3:** General characteristics of *Thuja sutchuenensis* as compared with the other four species of *Thuja*.

Region	Species	Height	DBH	Age	Radial growth	Habitat and	Shade	Major associated	Source
		(m)	(cm)	(year)	(mm/year)	Altitude	tolerance	species as examples	
a	*T*. *sutchuenensis*	2‒8	3‒7	30–60	0.5‒1.1	Limestone cliffs,	Intolerant	*Quercus engleriana*	This
		(16)	(40)	(293)		steep slopes,		*Cyclobalanopsis oxyodon*	study
						and crest ridges		*Quercus spinosa*	
						(800) 1100‒		*Carpinus fargesiana*	
						2100 m		*Fagus engleriana*	
								*Tsuga chinensis*	
								*Pinus tabuliformis* var.	
								*henryi*	
								*Torreya fargesii*	
b	*T*. *koraiensis*	3‒10	10‒30	Not	Not available	Humid sites in	Not available	*Abies nephrolepis*	[29, 43]
			(80)	available		valleys, mountain		*Taxus cuspidata*	
						slopes and ridges,		*Pinus pumila*	
						sometimes in		*Quercus mongolica*	
						crevices of rocks		*Betula ermanii*	
	* *					400–1900 m		*Acer ukurunduense*	
c	*T*. *standishii*	15‒30	40‒60	Not	c. 1.4‒2.3	Mountains	Tolerant	*Abies firma*	[44, 45, 46]
		(35)	(100)	available		Rocky or		*Abies homolepis*	
						Steep slopes		*Tsuga sieboldii*	
						Moist, mesic, and		*Tsuga diversifolia*	
						xeric sites		*Pinus parviflora*	
						(250) 1000‒		*Thujopsis dolabrata*	
						2000 (2150) m		*Fagus crenata*	
d	*T*. *occidentialis*	12‒15	30‒60	Cliff:	Limestone	Dry, mesic and	Tolerant, and	*Abies balsamea*	[39, 40, 47, 48]
		(38)	(175)	200–500	cliffs:	wet sites	intermediate	*Larix laricina*	[49, 50]
		Stunted or		(1032)	0.05‒0.17	limestone cliffs,	Stress-	*Picea mariana*	[51]
		prostrate		Non-cliff:	Acadian	swamps and	tolerant	*Tsuga canadensis*	
		in harsh		80	forest:	abandoned	Occurring at	*Pinus banksiana*	
		limestone		(400)	0.43‒0.82	pasture sites	All stages of	*Pinus strobus*	
		cliffs			Bogs: 1.55	(10) 150‒	forest	*Acer rubrum*	
						600 (670) m	succession	*Betula papyrifera*	
								*Populus balsamifera*	
e	*T*. *plicata*	40‒50	100‒300		10‒20	Gentle and	Tolerant	*Tsuga heterophylla*	[52, 53]
		(60)	(584)	(1460)	on the best	steep slopes	Occurring at	*Pseudotsuga menziesii*	[54, 55]
					moist sites	with moist soils,	all stages of	*Abies amabilis*	[56]
					But 200 years	rocky slopes	forest	*Taxus brevifolia*	
					can be	and river valleys	succession	*Abies grandis*	
					7.6 cm dbh	0–1500 (2200) m		*Pinus monticola*	

Common ranges of variables are presented with maximum values in parentheses. a. Daba Mountains, southwestern China; b. Korea and Changbei Mountains at the boundary between Korea and northeastern China; c. Honshu and Shikoku, Japan; d. Eastern North America; e. Western North America

The life history characteristics of *T*. *sutchuenensis* are similar to those of living fossil plants of the Yangtze River valley that are found in unstable and harsh habitats where natural disturbances occur frequently, and there is little competition from other species,as exemplified by *Ginkgo biloba* in the limestone habitat of the Dalou Mountains [14], *Metasequoia glyptostroboides* in wet lower mountain slopes and stream valleys in the border region of Hubei and Hunan provinces and Chongqing Municipality [13], *Davidia involucrata* on scree slopes or valley slopes and in valley bottoms, where the soil often contains much gravel, on Mt. Emei, Sichuan [12], and *Cathaya argyrophylla* on the steep topography of Mt. Jinfo [62].

### Implications for Conservation

Efforts to conserve *T*. *sutchuenensis* will focus on the individuals in their natural habitats. Cliffs, forest margins, canopy gaps and roadsides in the study sites provide micro-habitats favorable to the regeneration of this light-demanding species. Saplings on roadsides, being especially vulnerable, should be saved where practicable. A genetic study showed that *T*. *sutchuenensis* populations have a relatively high level of within-population genetic diversity despite the small number of individuals [63]. Conservation measures should also be directly towards large numbers of seedlings *in situ*, which will require management and cooperation from local communities. *In situ* efforts to conserve remaining habitats need to be combined with *ex situ* research on seed propagation, with a view to establishing a new generation of plants both in cultivation and in the wild [63]. According to Jin [64], *T*. *sutchuenensis* can establish roots from cutting shoots, indicating that this species is able to be propagated by clonal reproduction. Cuttage breeding may provide the most effective artificial propagation for the species.

Demand for use of this species far outweighs current conservation efforts to replenish the supply. For a long time, *T*. *sutchuenensis* has been a preferred wood for the construction of houses, fences, and pigpens by local people because of its resistance to rotting. It is used in arts or crafts such as root carving and bonsai because of its flexibility. Better management and effective conservation are needed to ensure the recruitment and continuing survival of this rare and endangered Tertiary relict species in its natural habitats.

## Supporting Information

S1 Table Woody species (RBA > 0.01%) occurring in each community type, and their DBH- and height-class frequency distributions. (PDF)
