# Supplementary material for: Community Structure and Survival of Tertiary Relict Thuja sutchuenensis (Cupressaceae) in the Subtropical Daba Mountains, Southwestern China
Source: PLoS One. 2015 Apr 30;10(4):e0125307. doi: 10.1371/journal.pone.0125307 (PMC4415794; doi:10.1371/journal.pone.0125307)
Supplement: S1 Table — (PDF) [file pone.0125307.s001.pdf]

S1 Table Woody species (RBA > 0.01%) occurring in each community type, and their DBH- and height-class frequency distributions.

Type A— the plant community on cliffs: 10 plots (Plot 3, Plot 4, Plot 7, Plot 8, Plot 9, Plot 10, Plot 12, Plot 17, Plot 19, Plot 20)

Total area of the 10 plots: 2550.0 m^2

(S): sprouts; ¥: absence

Species	DBH Mean (cm)	DBH Max. (cm)	No. of Stems N	DBH Class 0~5	5~10	10~15	15~20	20~25	25~30	30~35	35~40 (cm)	Height Class 1.3~2	2~4	4~6	6~8	8~10	10~12	12~14	14~16 (m)
1 *Thuja sutchuenensis*	11.3	40.0	225	57	55	49	34	21	4	3	2	42	77	58	25	10	5	5	3
(S) *Thuja sutchuenensis*	8.6	16.0	22	5	7	8	2	¥	¥	¥	¥	6	6	10	¥	¥	¥	¥	¥
2 *Quercus engleriana*	8.5	28.0	18	1	10	6	¥	¥	1	¥	¥	¥	1	16	¥	1	¥	¥	¥
3 *Quercus dolicholep*	9.3	20.0	12	4	4	¥	3	1	¥	¥	¥	¥	4	4	4	¥	¥	¥	¥
(S) *Quercus dolicholepis*	8.0	8.0	3	¥	3	¥	¥	¥	¥	¥	¥	¥	¥	3	¥	¥	¥	¥	¥
4 *Cyclobalanopsis oxyodon*	7.6	10.0	17	6	11	¥	¥	¥	¥	¥	¥	¥	5	6	6	¥	¥	¥	¥
6 *Quercus phillyreoides*	7.2	8.0	16	2	14	¥	¥	¥	¥	¥	¥	¥	13	3	¥	¥	¥	¥	¥
7 *Platycarya strobilacea*	3.7	5.0	43	35	8	¥	¥	¥	¥	¥	¥	¥	30	13	¥	¥	¥	¥	¥
8 *Daphniphyllum macropodum*	7.3	12.0	8	4	¥	4	¥	¥	¥	¥	¥	¥	3	1	¥	4	¥	¥	¥
9 *Quercus spinosa*	6.2	10.0	8	5	3	¥	¥	¥	¥	¥	¥	¥	8	¥	¥	¥	¥	¥	¥
10 *Buxus microphylla* subsp. *sinica*	2.6	3.0	54	54	¥	¥	¥	¥	¥	¥	¥	10	44	¥	¥	¥	¥	¥	¥
11 *Symplocos multipes*	12.0	12.0	2	¥	¥	2	¥	¥	¥	¥	¥	¥	¥	¥	2	¥	¥	¥	¥
12 *Zanthoxylum ovalifolium*	4.3	5.0	11	4	7	¥	¥	¥	¥	¥	¥	¥	11	¥	¥	¥	¥	¥	¥
13 *Betula austrosinensis*	7.3	10.0	3	¥	2	1	¥	¥	¥	¥	¥	¥	¥	2	1	¥	¥	¥	¥
(S) *Betula austrosinensis*	6.0	6.0	1	¥	1	¥	¥	¥	¥	¥	¥	¥	¥	1	¥	¥	¥	¥	¥
14 *Cornus oblonga*	5.6	8.0	5	¥	5	¥	¥	¥	¥	¥	¥	¥	4	¥	1	¥	¥	¥	¥
15 *Ficus heteromorpha*	3.2	8.0	11	10	1	¥	¥	¥	¥	¥	¥	¥	11	¥	¥	¥	¥	¥	¥
16 *Pinus kwangtungensis*	4.4	6.0	5	3	2	¥	¥	¥	¥	¥	¥	¥	4	1	¥	¥	¥	¥	¥
17 *Emmenopterys henryi*	7.0	8.0	2	¥	2	¥	¥	¥	¥	¥	¥	¥	¥	1	1	¥	¥	¥	¥
18 *Lindera megaphylla*	4.0	4.0	6	6	¥	¥	¥	¥	¥	¥	¥	¥	6	¥	¥	¥	¥	¥	¥
19 *Cyclobalanopsis myrsinaefolia*	5.0	6.0	3	1	2	¥	¥	¥	¥	¥	¥	¥	2	1	¥	¥	¥	¥	¥
20 *Neocinnamomum fargesii*	2.1	2.5	17	17	¥	¥	¥	¥	¥	¥	¥	¥	17	¥	¥	¥	¥	¥	¥
21 *Toxicodendron succedaneum*	2.3	2.5	12	12	¥	¥	¥	¥	¥	¥	¥	¥	12	¥	¥	¥	¥	¥	¥
22 *Cyclobalanopsis multinervis*	8.0	8.0	1	¥	1	¥	¥	¥	¥	¥	¥	¥	¥	1	¥	¥	¥	¥	¥
23 *Betula utilis*	4.0	4.0	4	4	¥	¥	¥	¥	¥	¥	¥	¥	4	¥	¥	¥	¥	¥	¥
24 *Pittosporum truncatum*	3.4	5.0	5	4	1	¥	¥	¥	¥	¥	¥	¥	5	¥	¥	¥	¥	¥	¥
25 *Viburnum atrocyaneum*	2.7	3.0	7	7	¥	¥	¥	¥	¥	¥	¥	1	6	¥	¥	¥	¥	¥	¥
26 *Euptelea pleiosperma*	3.0	3.0	6	6	¥	¥	¥	¥	¥	¥	¥	¥	6	¥	¥	¥	¥	¥	¥
27 *Cephalotaxus fortunei*	3.0	3.0	6	6	¥	¥	¥	¥	¥	¥	¥	¥	6	¥	¥	¥	¥	¥	¥
28 *Stranvaesia davidiana*	2.6	3.0	7	7	¥	¥	¥	¥	¥	¥	¥	¥	7	¥	¥	¥	¥	¥	¥

29 *Padus wilsonii*	4.0	4.0	3	3	¥	¥	¥	¥	¥	¥	¥	¥	3	¥	¥	¥	¥	¥	¥
30 *Rhus chinensis*	4.0	4.0	3	3	¥	¥	¥	¥	¥	¥	¥	¥	3	¥	¥	¥	¥	¥	¥
31 *Elaeagnus difficilis*	3.0	3.0	5	5	¥	¥	¥	¥	¥	¥	¥	¥	5	¥	¥	¥	¥	¥	¥
32 *Tsuga chinensis*	3.5	4.0	2	2	¥	¥	¥	¥	¥	¥	¥	¥	2	¥	¥	¥	¥	¥	¥
33 *Pinus tabuliformis* var. *henryi*	3.6	6.0	2	1	1	¥	¥	¥	¥	¥	¥	¥	1	¥	¥	¥	¥	¥	¥
34 *Juniperus formosana*	6.0	6.0	1	¥	1	¥	¥	¥	¥	¥	¥	¥	1	¥	¥	¥	¥	¥	¥
35 *Cladrastis delavayi*	6.0	6.0	1	¥	1	¥	¥	¥	¥	¥	¥	¥	¥	¥	1	¥	¥	¥	¥
36 *Osmanthus armatus*	3.2	4.0	3	3	¥	¥	¥	¥	¥	¥	¥	¥	3	¥	¥	¥	¥	¥	¥
37 *Decaisnea insignis*	4.0	4.0	2	2	¥	¥	¥	¥	¥	¥	¥	¥	2	¥	¥	¥	¥	¥	¥
38 *Viburnum betulifolium*	2.6	3.0	4	4	¥	¥	¥	¥	¥	¥	¥	3	1	¥	¥	¥	¥	¥	¥
39 *Phoebe bournei*	1.5	1.5	12	12	¥	¥	¥	¥	¥	¥	¥	2	10	¥	¥	¥	¥	¥	¥
40 *Salix wallichiana*	3.0	3.0	3	3	¥	¥	¥	¥	¥	¥	¥	¥	3	¥	¥	¥	¥	¥	¥
41 *Coriaria nepalensis*	3.0	3.0	3	3	¥	¥	¥	¥	¥	¥	¥	¥	3	¥	¥	¥	¥	¥	¥
42 *Acer davidii*	2.0	2.5	6	6	¥	¥	¥	¥	¥	¥	¥	¥	6	¥	¥	¥	¥	¥	¥
43 *Dalbergia hancei*	1.9	2.0	7	7	¥	¥	¥	¥	¥	¥	¥	¥	7	¥	¥	¥	¥	¥	¥
44 *Rhododendron adenopodum*	1.9	3.5	5	5	¥	¥	¥	¥	¥	¥	¥	¥	5	¥	¥	¥	¥	¥	¥
(S) *Rhododendron adenopodum*	1.5	1.5	3	3	¥	¥	¥	¥	¥	¥	¥	¥	3	¥	¥	¥	¥	¥	¥
45 *Sageretia* sp.	2.3	2.5	4	4	¥	¥	¥	¥	¥	¥	¥	¥	4	¥	¥	¥	¥	¥	¥
46 *Torreya fargesii*	2.3	3.0	3	3	¥	¥	¥	¥	¥	¥	¥	2	1	¥	¥	¥	¥	¥	¥
47 *Cinnamomum mairei*	4.0	4.0	1	1	¥	¥	¥	¥	¥	¥	¥	¥	1	¥	¥	¥	¥	¥	¥
48 *Pieris japonica*	2.0	2.0	4	4	¥	¥	¥	¥	¥	¥	¥	¥	¥	4	¥	¥	¥	¥	¥
49 *Premna microphylla*	1.9	2.0	4	4	¥	¥	¥	¥	¥	¥	¥	1	3	¥	¥	¥	¥	¥	¥
50 *Taxus wallichiana* var. *chinensis*	2.0	2.0	3	3	¥	¥	¥	¥	¥	¥	¥	3	¥	¥	¥	¥	¥	¥	¥
51 *Itea oblonga*	3.0	3.0	1	1	¥	¥	¥	¥	¥	¥	¥	¥	1	¥	¥	¥	¥	¥	¥
52 *Viburnum oliganthum*	3.0	3.0	1	1	¥	¥	¥	¥	¥	¥	¥	1	¥	¥	¥	¥	¥	¥	¥
53 *Rubus wallichianus*	3.0	3.0	1	1	¥	¥	¥	¥	¥	¥	¥	¥	1	¥	¥	¥	¥	¥	¥

Type B— the plant community on steep slopes: 10 plots (Plot 1, Plot 2, Plot 11, Plot 13, Plot 14, Plot 15, Plot 16, Plot 18, Plot 21, Plot 22)

Total area of the 10 plots: 3200.0 m^2

(S): sprouts; ¥: absence

Species	DBH Mean (cm)	DBH Max. (cm)	No. of Stems N	DBH Class 0~5	5~10	10~15	15~20	20~25	25~30	30~35	35~40 (cm)	Height Class 1.3~2	2~4	4~6	6~8	8~10	10~12	12~14	14~16 (m)
1 *Thuja sutchuenensis*	15.0	35.0	155	32	55	31	16	17	2	2	¥	12	47	41	15	22	6	6	6
(S) *Thuja sutchuenensis*	8.9	27.0	28	6	10	8	3	¥	1	¥	¥	4	9	8	4	3	¥	¥	¥
2 *Quercus engleriana*	21.5	35.0	13	¥	¥	4	1	3	3	1	1	¥	¥	¥	¥	7	1	1	4
3 *Cyclobalanopsis oxyodon*	14.2	20.0	40	¥	¥	8	29	1	¥	2	¥	3	12	7	12	4	¥	¥	2
(S) *Cyclobalanopsis oxyodon*	2.0	2.0	2	2	¥	¥	¥	¥	¥	¥	¥	2	¥	¥	¥	¥	¥	¥	¥
4 *Carpinus fargesiana*	6.9	20.0	39	18	14	4	2	1	¥	¥	¥	¥	22	6	¥	3	7	¥	1
(S) Carpinus fargesiana	9.0	9.0	3	¥	3	¥	¥	¥	¥	¥	¥	¥	¥	¥	¥	¥	3	¥	¥
5 *Fagus engleriana*	10.4	18.0	18	5	1	11	1	¥	¥	¥	¥	3	1	2	¥	1	2	9	¥
(S) *Fagus engleriana*	5.4	12.0	8	5	1	2	¥	¥	¥	¥	¥	3	1	2	¥	1	1	¥	¥

6 *Torreya fargesii*	7.3	28.0	26	13	10	¥	¥	2	1	¥	¥	1	20	3	2	¥	¥	¥	¥
(S)*Torreya fargesii*	7.0	7.0	5	¥	5	¥	¥	¥	¥	¥	¥	¥	5	¥	¥	¥	¥	¥	¥
7 *Cyclobalanopsis myrsinaefolia*	11.4	30.0	10	2	4	2	¥	¥	¥	2	¥	¥	4	4	¥	¥	¥	¥	2
8 *Platycarya strobilacea*	7.5	12.0	35	5	29	1	¥	¥	¥	¥	¥	¥	7	6	5	17	¥	¥	¥
(S) *Platycarya strobilacea*	9.0	9.0	15	¥	15	¥	¥	¥	¥	¥	¥	¥	¥	¥	¥	15	¥	¥	¥
9 *Cornus oblonga*	9.7	22.0	16	¥	10	1	4	1	¥	¥	¥	¥	2	¥	9	¥	¥	¥	5
10 *Juglans cathayensis*	13.5	40.0	4	2	1	¥	¥	¥	¥	¥	1	¥	2	1	¥	¥	1	¥	¥
11 *Daphniphyllum macropodum*	7.2	18.0	13	8	¥	2	3	¥	¥	¥	¥	¥	8	4	¥	¥	¥	¥	1
12 *Tsuga chinensis*	13.8	16.0	5	¥	¥	4	1	¥	¥	¥	¥	¥	¥	¥	¥	2	¥	2	1
13 *Lithocarpus rosthornii*	6.4	16.0	16	6	7	2	1	¥	¥	¥	¥	1	5	8	2	¥	¥	¥	¥
14 Quercus spinosa	8.9	25.0	7	3	1	2	¥	1	¥	¥	¥	¥	4	1	1	1	¥	¥	¥
15 *Taxus wallichiana var. chinensis*	7.0	18.0	7	4	1	¥	2	¥	¥	¥	¥	3	3	1	¥	¥	¥	¥	¥
16 *Sorbus wilsoniana*	14.0	25.0	2	1	¥	¥	¥	¥	1	¥	¥	¥	2	¥	¥	¥	¥	¥	¥
17 *Illicium micranthum*	9.6	14.0	5	2	¥	3	¥	¥	¥	¥	¥	1	1	¥	¥	3	¥	¥	¥
18 *Pinus tabuliformis* var. *henryi*	9.3	16.0	4	1	1	1	1	¥	¥	¥	¥	1	¥	¥	2	1	¥	¥	¥
19 *Quercus phillyreoides*	8.8	9.0	6	¥	6	¥	¥	¥	¥	¥	¥	¥	¥	6	¥	¥	¥	¥	¥
20 *Symplocos multipes*	9.3	12.0	4	1	¥	3	¥	¥	¥	¥	¥	1	¥	¥	3	¥	¥	¥	¥
21 *Quercus dolicholepis*	5.2	8.0	15	3	12	¥	¥	¥	¥	¥	¥	¥	3	12	¥	¥	¥	¥	¥
22 *Ilex* sp.	6.5	12.0	8	1	5	2	¥	¥	¥	¥	¥	¥	2	5	1	¥	¥	¥	¥
23 *Machilus* sp.	13.0	16.0	2	¥	¥	1	1	¥	¥	¥	¥	¥	¥	¥	2	¥	¥	¥	¥
24 *Viburnum atrocyaneum*	3.9	16.0	9	8	¥	¥	1	¥	¥	¥	¥	1	5	2	¥	¥	¥	¥	1
(S) *Viburnum atrocyaneum*	2.0	2.0	4	4	¥	¥	¥	¥	¥	¥	¥	1	2	1	¥	¥	¥	¥	¥
25 *Viburnum betulifolium*	4.7	16.0	5	4	¥	¥	1	¥	¥	¥	¥	¥	4	¥	¥	¥	¥	¥	1
26 *Emmenopterys henryi*	16.0	16.0	1	¥	¥	¥	1	¥	¥	¥	¥	¥	¥	¥	1	¥	¥	¥	¥
27 *Distylium myricoides*	7.3	12.0	3	1	1	1	¥	¥	¥	¥	¥	¥	1	¥	2	¥	¥	¥	¥
28 *Rhus punjabensis* var. *sinica*	5.7	14.0	3	2	¥	1	¥	¥	¥	¥	¥	¥	2	¥	1	¥	¥	¥	¥
(S) *Rhus punjabensis* var. *sinica*	1.5	1.5	1	1	¥	¥	¥	¥	¥	¥	¥	¥	1	¥	¥	¥	¥	¥	¥
29 *Acer maximowiczii*	7.0	7.0	4	¥	4	¥	¥	¥	¥	¥	¥	¥	¥	¥	¥	4	¥	¥	¥
(S) *Acer maximowiczii*	7.0	7.0	3	¥	3	¥	¥	¥	¥	¥	¥	¥	¥	¥	¥	3	¥	¥	¥
30 *Tilia tuan*	9.0	12.0	2	¥	1	1	¥	¥	¥	¥	¥	¥	¥	2	¥	¥	¥	¥	¥
31 *Betula utilis*	6.0	6.0	5	¥	5	¥	¥	¥	¥	¥	¥	¥	¥	5	¥	¥	¥	¥	¥
32 *Acer davidii*	3.5	6.0	13	10	3	¥	¥	¥	¥	¥	¥	¥	5	8	¥	¥	¥	¥	¥
(S) *Acer davidii*	3.0	3.0	5	5	¥	¥	¥	¥	¥	¥	¥	¥	¥	5	¥	¥	¥	¥	¥
33 *Zanthoxylum ovalifolium*	4.7	6.0	7	3	4	¥	¥	¥	¥	¥	¥	¥	7	¥	¥	¥	¥	¥	¥
(S) *Zanthoxylum ovalifolium*	3.0	3.0	2	2	¥	¥	¥	¥	¥	¥	¥	¥	2	¥	¥	¥	¥	¥	¥
34 *Cyclobalanopsis multinervis*	6.0	8.0	4	1	3	¥	¥	¥	¥	¥	¥	¥	1	3	¥	¥	¥	¥	¥
35 *Metapanax davidii*	3.1	8.0	13	12	1	¥	¥	¥	¥	¥	¥	1	11	¥	1	¥	¥	¥	¥
(S) Metapanax davidii	2.5	2.5	4	4	¥	¥	¥	¥	¥	¥	¥	¥	4	¥	¥	¥	¥	¥	¥
36 *Osmanthus yunnanensis*	3.5	6.0	8	4	4	¥	¥	¥	¥	¥	¥	4	¥	4	¥	¥	¥	¥	¥
37 *Meliosma flexuosa*	6.8	12.0	2	1	¥	1	¥	¥	¥	¥	¥	1	¥	¥	1	¥	¥	¥	¥
38 *Sorbus xanthoneura*	12.0	12.0	1	¥	¥	1	¥	¥	¥	¥	¥	¥	¥	¥	1	¥	¥	¥	¥
39 *Stranvaesia davidiana*	12.0	12.0	1	¥	¥	1	¥	¥	¥	¥	¥	¥	1	¥	¥	¥	¥	¥	¥
40 Betula albosinensis	12.0	12.0	1	¥	¥	1	¥	¥	¥	¥	¥	¥	¥	¥	1	¥	¥	¥	¥
41 *Euonymus myrianthus*	6.3	8.0	3	1	2	¥	¥	¥	¥	¥	¥	¥	1	2	¥	¥	¥	¥	¥
42 *Viburnum sympodiale*	8.0	8.0	2	¥	2	¥	¥	¥	¥	¥	¥	¥	¥	2	¥	¥	¥	¥	¥
43 *Rhododendron* sp.	3.9	8.0	5	3	2	¥	¥	¥	¥	¥	¥	1	2	2	¥	¥	¥	¥	¥
44 *Pinus armandii*	6.0	6.0	3	¥	3	¥	¥	¥	¥	¥	¥	¥	¥	3	¥	¥	¥	¥	¥
45 *Diospyros kaki* var. *silvestris*	6.0	10.0	2	1	¥	1	¥	¥	¥	¥	¥	¥	1	1	¥	¥	¥	¥	¥
46 *Tilia nanchuanensis*	10.0	10.0	1	¥	¥	1	¥	¥	¥	¥	¥	¥	¥	¥	¥	1	¥	¥	¥

Species	DBH Mean (cm)	DBH Max. (cm)	No. of Stems N	DBH 0~5	5~10	10~15	15~20	20~25	25~30	30~35	35~40 (cm)	Height 1.3~2	2~4	4~6	6~8	8~10	10~12	12~14	14~16 (m)
47 *Euptelea pleiosperma*	4.3	6.0	4	2	2	¥	¥	¥	¥	¥	¥	¥	2	2	¥	¥	¥	¥	¥
48 *Cinnamomum* sp.	2.1	3.5	19	19	¥	¥	¥	¥	¥	¥	¥	1	17	1	¥	¥	¥	¥	¥
49 *Cladrastis delavayi*	6.0	6.0	2	¥	2	¥	¥	¥	¥	¥	¥	¥	¥	1	1	¥	¥	¥	¥
50 *Cercis glabra*	5.3	8.0	2	1	1	¥	¥	¥	¥	¥	¥	¥	¥	1	1	¥	¥	¥	¥
51 *Vaccinium japonicum* var. *sinicum*	8.0	8.0	1	¥	1	¥	¥	¥	¥	¥	¥	¥	¥	¥	¥	1	¥	¥	¥
52 *Cornus controversa*	8.0	8.0	1	¥	1	¥	¥	¥	¥	¥	¥	¥	1	¥	¥	¥	¥	¥	¥
53 *Coriaria nepalensis*	8.0	8.0	1	¥	1	¥	¥	¥	¥	¥	¥	¥	1	¥	¥	¥	¥	¥	¥
54 *Corylus chinensis*	8.0	8.0	1	¥	1	¥	¥	¥	¥	¥	¥	¥	1	¥	¥	¥	¥	¥	¥
55 *Cephalotaxus fortunei*	3.8	6.0	4	3	1	¥	¥	¥	¥	¥	¥	¥	4	¥	¥	¥	¥	¥	¥
56 *Toxicodendron succedaneum*	5.0	6.0	2	1	1	¥	¥	¥	¥	¥	¥	¥	1	¥	1	¥	¥	¥	¥
57 *Cotoneaster salicifolius*	3.7	6.0	3	2	1	¥	¥	¥	¥	¥	¥	¥	¥	3	¥	¥	¥	¥	¥
(S) *Cotoneaster salicifolius*	2.5	3.0	2	2	¥	¥	¥	¥	¥	¥	¥	¥	¥	2	¥	¥	¥	¥	¥
58 *Ficus heteromorpha*	3.7	6.0	3	2	1	¥	¥	¥	¥	¥	¥	¥	3	¥	¥	¥	¥	¥	¥
59 *Clethra fargesii*	7.0	7.0	1	¥	1	¥	¥	¥	¥	¥	¥	¥	1	¥	¥	¥	¥	¥	¥
60 *Betula luminifera*	4.0	4.0	3	3	¥	¥	¥	¥	¥	¥	¥	¥	¥	3	¥	¥	¥	¥	¥
61 *Toricellia angulata*	4.5	6.0	2	1	1	¥	¥	¥	¥	¥	¥	¥	1	¥	1	¥	¥	¥	¥
62 *Pieris japonica*	3.5	5.0	3	2	1	¥	¥	¥	¥	¥	¥	¥	3	¥	¥	¥	¥	¥	¥
63 *Ilex bioritsensis*	2.3	6.0	4	3	1	¥	¥	¥	¥	¥	¥	3	¥	1	¥	¥	¥	¥	¥
64 *Salix* sp.	6.0	6.0	1	¥	1	¥	¥	¥	¥	¥	¥	¥	¥	1	¥	¥	¥	¥	¥
65 *Hydrangea rosthornii*	6.0	6.0	1	¥	1	¥	¥	¥	¥	¥	¥	¥	¥	1	¥	¥	¥	¥	¥
66 *Pterostyrax psilophyllus*	6.0	6.0	1	¥	1	¥	¥	¥	¥	¥	¥	¥	¥	1	¥	¥	¥	¥	¥
67 *Lindera megaphylla*	6.0	6.0	1	¥	1	¥	¥	¥	¥	¥	¥	¥	¥	1	¥	¥	¥	¥	¥
68 *Betula austrosinensis*	6.0	6.0	1	¥	1	¥	¥	¥	¥	¥	¥	¥	¥	1	¥	¥	¥	¥	¥
69 *Symplocos ernestii*	4.0	4.0	2	2	¥	¥	¥	¥	¥	¥	¥	¥	2	¥	¥	¥	¥	¥	¥
70 *Dalbergia hancei*	2.1	3.0	6	6	¥	¥	¥	¥	¥	¥	¥	¥	1	5	¥	¥	¥	¥	¥
71 *Hydrangea strigosa*	2.5	4.0	3	3	¥	¥	¥	¥	¥	¥	¥	¥	3	¥	¥	¥	¥	¥	¥
72 *Sorbus keissleri*	2.8	4.0	2	2	¥	¥	¥	¥	¥	¥	¥	¥	2	¥	¥	¥	¥	¥	¥
73 *Zanthoxylum simulans*	4.0	4.0	1	1	¥	¥	¥	¥	¥	¥	¥	¥	1	¥	¥	¥	¥	¥	¥
74 *Cinnamomum mairei*	4.0	4.0	1	1	¥	¥	¥	¥	¥	¥	¥	¥	¥	1	¥	¥	¥	¥	¥
75 *Alangium chinense*	4.0	4.0	1	1	¥	¥	¥	¥	¥	¥	¥	¥	1	¥	¥	¥	¥	¥	¥
76 *Pittosporum truncatum*	2.2	3.0	3	3	¥	¥	¥	¥	¥	¥	¥	1	2	¥	¥	¥	¥	¥	¥
77 *Stachyurus himalaicus*	2.5	2.5	2	2	¥	¥	¥	¥	¥	¥	¥	¥	2	¥	¥	¥	¥	¥	¥
78 *Pinus kwangtungensis*	2.0	2.0	3	3	¥	¥	¥	¥	¥	¥	¥	1	2	¥	¥	¥	¥	¥	¥
(S) *Pinus kwangtungensis*	2.0	2.0	2	2	¥	¥	¥	¥	¥	¥	¥	1	1	¥	¥	¥	¥	¥	¥

Type C— the plant communities on crest ridges: 5 plots (Plot5, Plot6, Plot23, Plot24, Plot25)

Total area of the 5 plots: 1200.0 m^2

(S): sprouts; ¥: absence

Species	DBH Mean (cm)	DBH Max. (cm)	No. of Stems N	DBH Class 0~5	5~10	10~15	15~20	20~25	25~30	30~35	35~40 (cm)	Height Class 1.3~2	2~4	4~6	6~8	8~10	10~12	12~14	14~16 (m)
1 *Thuja sutchuenensis*	5.8	11.1	60	8	17	11	22	2	¥	¥	¥	¥	11	26	10	11	2	¥	¥

(S) *Thuja sutchuenensis*	8.0	16.0	9	4	1	2	2	¥	¥	¥	¥	¥	5	3	1	¥	¥	¥	¥
2 *Tsuga chinensis*	11.7	16.0	39	¥	12	17	10	¥	¥	¥	¥	¥	10	¥	2	2	25	¥	¥
3 *Quercus spinosa*	12.5	25.0	22	¥	11	6	1	¥	4	¥	¥	¥	¥	11	4	3	4	¥	¥
(S) *Quercus spinosa*	12.0	12.0	5	¥	¥	5	¥	¥	¥	¥	¥	¥	¥	¥	2	2	¥	¥	¥
4 *Torreya fargesii*	12.8	18.0	6	¥	1	3	2	¥	¥	¥	¥	¥	¥	3	3	¥	¥	¥	¥
5 *Carpinus fargesiana*	5.4	8.0	17	6	11	¥	¥	¥	¥	¥	¥	¥	15	2	¥	¥	¥	¥	¥
6 *Cyclobalanopsis oxyodon*	5.0	5.0	14	¥	14	¥	¥	¥	¥	¥	¥	¥	14	¥	¥	¥	¥	¥	¥
(S) *Cyclobalanopsis oxyodon*	5.0	5.0	10	¥	10	¥	¥	¥	¥	¥	¥	¥	10	¥	¥	¥	¥	¥	¥
7 *Buxus microphylla* subsp. *sinica*	4.2	6.0	16	9	7	¥	¥	¥	¥	¥	¥	1	15	¥	¥	¥	¥	¥	¥
8 *Rhododendron* sp.	3.9	6.0	16	9	7	¥	¥	¥	¥	¥	¥	¥	9	¥	7	¥	¥	¥	¥
9 *Betula austrosinensis*	4.8	5.0	11	1	10	¥	¥	¥	¥	¥	¥	1	10	¥	¥	¥	¥	¥	¥
10 *Sorbus glomerulata*	6.0	6.0	4	¥	4	¥	¥	¥	¥	¥	¥	¥	¥	4	¥	¥	¥	¥	¥
11 *Osmanthus yunnanensis*	3.0	3.0	13	¥	¥	¥	¥	¥	¥	¥	¥	¥	13	¥	¥	¥	¥	¥	¥
12 *Pinus kwangtungens*	2.3	3.0	8	8	¥	¥	¥	¥	¥	¥	¥	¥	8	¥	¥	¥	¥	¥	¥
13 *Viburnum betulifolium*	1.5	1.5	7	7	¥	¥	¥	¥	¥	¥	¥	¥	7	¥	¥	¥	¥	¥	¥
14 *Acer davidii*	1.5	1.5	2	2	¥	¥	¥	¥	¥	¥	¥	¥	2	¥	¥	¥	¥	¥	¥
